# Did extreme nest predation favor the evolution of obligate brood parasitism in a duck?

**DOI:** 10.1002/ece3.9251

**Published:** 2022-09-19

**Authors:** Bruce E. Lyon, Alejandra Carminati, Geneviève Goggin, John M. Eadie

**Affiliations:** ^1^ Department of Ecology and Evolutionary Biology University of California Santa Cruz California USA; ^2^ Ciudad Autónoma de Buenos Aires Argentina; ^3^ Vancouver British Columbia Canada; ^4^ Department of Wildlife, Fish, and Conservation Biology University of California, Davis Davis California USA

**Keywords:** artificial nest experiment, black‐headed duck, brood parasitism, cost of parasitism, evolution obligate parasitism, *Heteronetta*, host, life history, nest predation, nest survival

## Abstract

Obligate brood parasites depend entirely on other species to raise their offspring. Most avian obligate brood parasites have altricial offspring that require enormous amounts of posthatching parental care, and the large fecundity boost that comes with complete emancipation from parental care likely played a role in the independent evolution of obligate parasitism in several altricial lineages. The evolution of obligate parasitism in the black‐headed duck, however, is puzzling because its self‐feeding precocial offspring should not constrain parental fecundity of a potential brood parasite in the way that altricial offspring do. We used an experimental nest predation study to test the idea that high nest predation rates played a role in the evolution of brood parasitism in this enigmatic duck. Experimental duck eggs in untended nests suffered massive rapid predation, while eggs in tended nests of the three main hosts, all aggressive nest defenders, had very high success, illustrating the benefits of parasitizing these ‘bodyguard’ hosts.

## Graphical abstract

We used an experimental nest predation study to test the idea that high nest predation rates coupled with high success by laying in aggressive host nests played a role in the evolution of brood parasitism in the enigmatic black‐headed duck. Experimental duck eggs in untended nests suffered massive rapid predation, while eggs in tended nests of the three main hosts—all aggressive nest defenders—had very high success, illustrating the benefits of parasitizing these ‘bodyguard’ hosts.

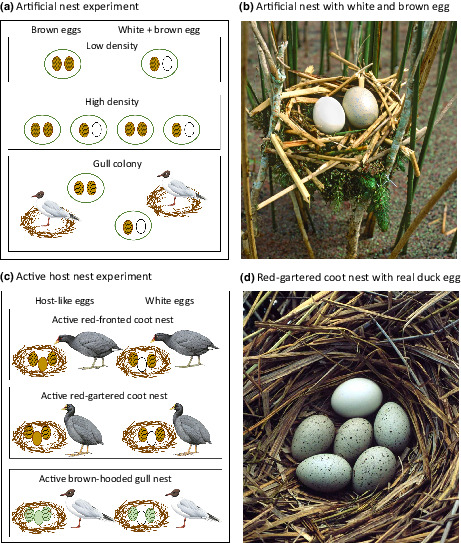

## 1 INTRODUCTION

Parental care has evolved independently many times (Clutton Brock, 1991) attesting to the benefits of providing care to offspring for many species. Species in a diversity of taxa have also evolved reproductive strategies that enable them to receive these benefits without paying the costs—for example, brood parasites manipulate other individuals to raise their offspring. Brood parasitism can occur within or among species. Conspecific brood parasites lay their eggs in the nests of other members of the same species (Field, 1992; Lyon & Eadie, 2008), while obligate brood parasites are completely parasitic and depend entirely on other species to raise their offspring. Obligate brood parasitism has evolved independently in a variety of taxonomic groups, including multiple times within birds (Davies, 2000; Rothstein, 1990) and social insects (Beibl et al., 2005; Bourke & Franks, 1995; d'Ettorre & Heinze, 2001; Hölldobler & Wilson, 1990), and once within fish (Sato, 1986).

Why some taxa have completely forsaken parental care, while others have not, is puzzling because the benefits from parasitism should apply to many species (Beibl et al., 2005; Bourke & Franks, 1991; Hamilton & Orians, 1965; Payne, 1977). Obligate brood parasitism is particularly well‐studied in birds, and two complementary approaches have been used to explore the factors that might have facilitated its evolution. First, some studies considered the ecological circumstances and proximate factors that might have favored laying eggs in nests other than their own (Hamilton & Orians, 1965; Krüger & Davies, 2002; Rothstein, 1993). A second demographic approach instead focuses on the lifetime fitness costs and benefits of laying eggs parasitically versus in a bird's own nest (Hamilton & Orians, 1965; Lyon & Eadie, 1991; Robert & Sorci, 2001; Yamauchi, 1995).

The evolution of brood parasitism is perhaps best studied in a life history evolution framework. The demographic theory approach to modeling life history evolution focuses on life history traits like fecundity and survival in age‐structured populations, and their contribution to fitness through effects on population growth (Reznick et al., 2002; Stearns, 1992). As a life history strategy, obligate parasitism will be favored when the lifetime fitness of obligate parasites exceeds the fitness of either purely parental individuals or facultative parasites that pursue both parasitism and parenting (Lyon & Eadie, 1991). This will depend on key parameters that influence fitness in age‐structured populations, including fecundity, juvenile survival, and adult survival. We previously suggested that fecundity could be a key demographic parameter for most evolutionary origins of avian obligate brood parasitism (Lyon & Eadie, 1991). Virtually, all avian obligate brood parasites have helpless altricial offspring that require extreme posthatching investment in the form of parental feedings. The eggs of altricial birds, in contrast, are thought to be cheap relative to the investment in offspring—in birds with inexpensive eggs but costly offspring, emancipation from parental care should enable a large increase in fecundity. Accordingly, if there are sufficient suitable hosts that can successfully raise those offspring, the massive fecundity boost from parasitism should favor complete emancipation from parental care in altricial birds once a species is able to successfully parasitize hosts of other species (Lyon & Eadie, 1991). Most previous work on the evolution of obligate brood parasitism has focused on the fecundity gains from brood parasitism, but it is also clear that brood parasites would avoid paying any survival costs that come with nesting.

**FIGURE 1:**
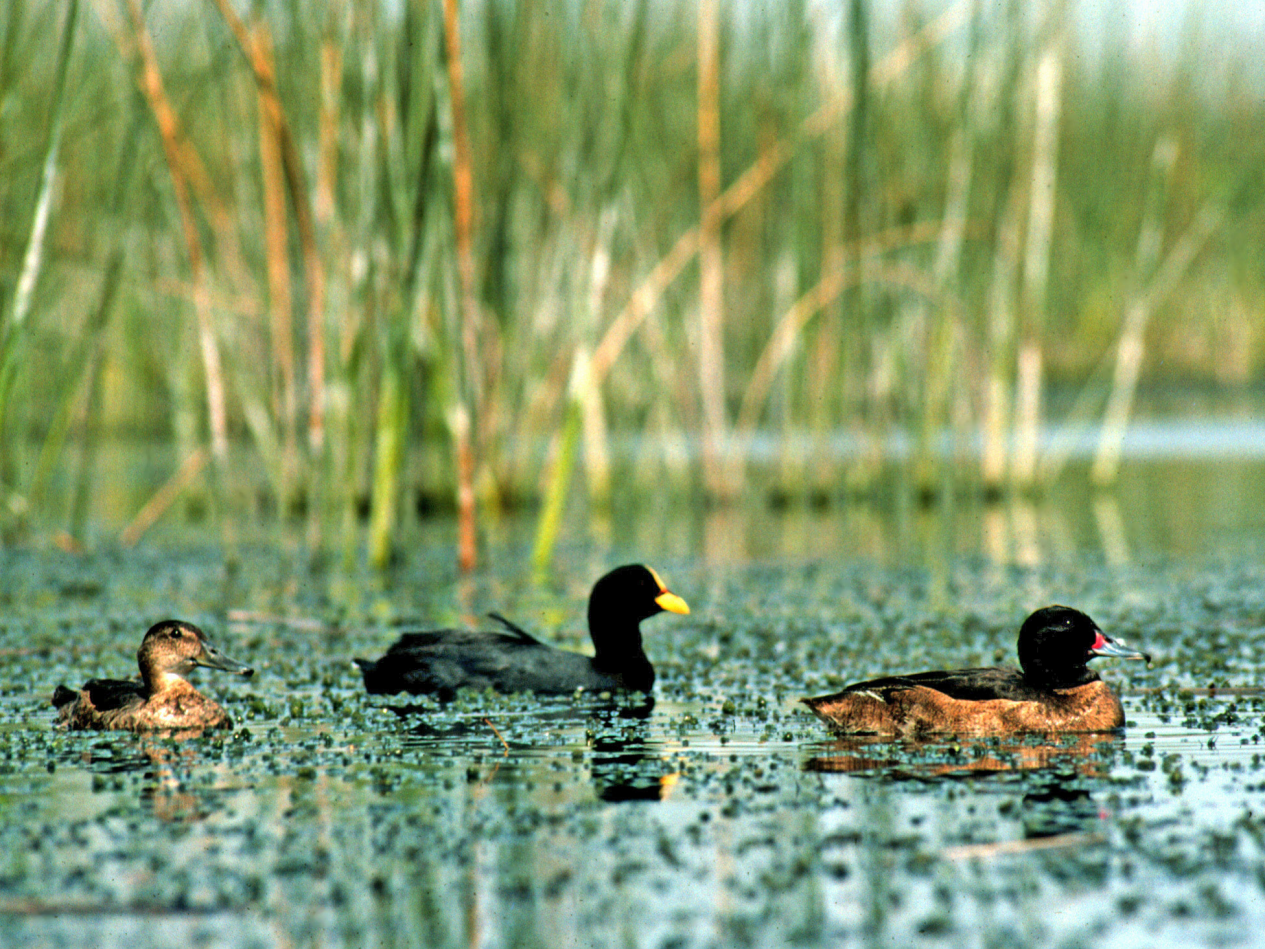
A pair of black‐headed ducks flanks a red‐gartered coot, their most important host species.

The black‐headed duck (*Heteronetta atricapilla*) of southern South America (Figure 1) is a glaring exception to this pattern—it is the only species of the 100 avian obligate brood parasites with precocial offspring (Davies, 2000; Lyon & Eadie, 1991; Weller, 1968). The ducklings not only feed themselves, but they also raise themselves after hatching without any parental care whatsoever (Lyon & Eadie, 2013; Weller, 1968). Why complete abandonment of parental care would be evolutionarily favored in a precocial species is puzzling. The large fecundity increase postulated for altricial parasites seems unlikely. Constraints on clutch size in precocial birds are unclear and debated (Ankney & MacInnes, 1978; Arnold & Rohwer, 1991; Winkler & Walters, 1983). The fact that precocial birds tend to have larger clutch sizes (Klomp, 1970; Lack, 1968) and more energy‐rich eggs than altricial birds (Rahn et al., 1975) suggests that precocial birds might not gain nearly as large a fecundity enhancement from complete emancipation from parental care as altricial birds (Lyon & Eadie, 1991). Thus, some other demographic variable must have been important in the evolution of brood parasitism in this unusual duck.

We propose that nest predation could have been important in the evolution of brood parasitism in this one species of precocial obligate brood parasite. Nest predation has been suggested as a proximate factor that triggers laying eggs in the nests of others, both for interspecific parasitism (Hamilton & Orians, 1965) and within‐species (conspecific) brood parasitism (Lyon & Eadie, 2008; McRae, 1997). It has not, however, received much attention as a potential demographic driver of parasitism in terms of fitness costs and benefits. Weller (1968) noted that the coot hosts (*Fulica* spp.) of the black‐headed ducks have high nesting success, which could be a factor. In our own study of the black‐headed duck, we also noted that the three main hosts used by the ducks—two species of coots and a gull—are all species that aggressively defend their nests against predators. We hypothesized that these aggressive hosts might have higher nesting success than black‐headed duck would, were the ducks to nest on their own in these predator‐rich wetlands (Lyon & Eadie, 2013). According to this hypothesis, the hosts function as protectors—effectively bodyguards—for the ducks' eggs, and an increase in nest success and hatching success, rather than solely an increase in fecundity, could have facilitated the evolution of obligate parasitism in this duck.

**FIGURE 2:**
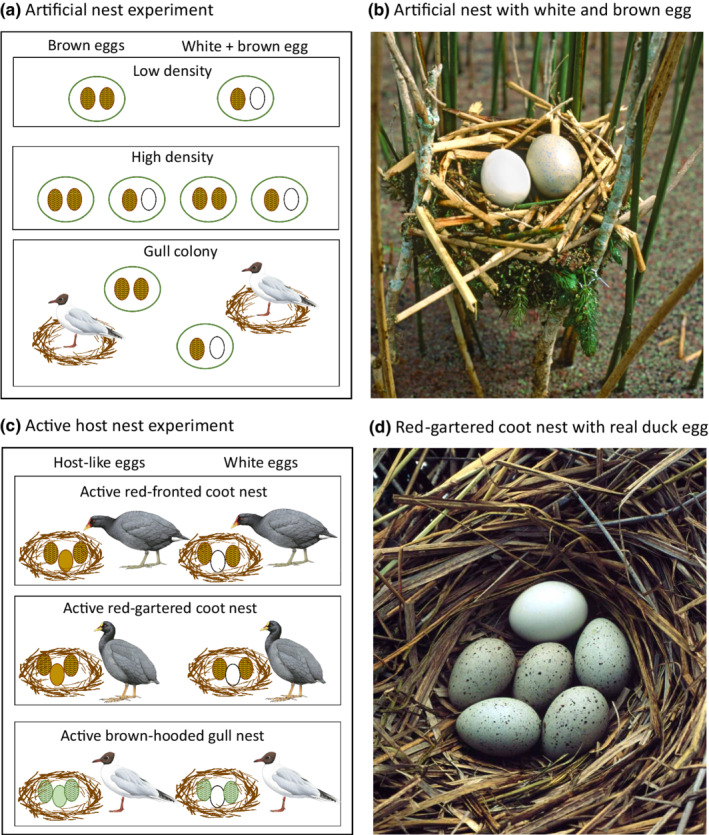
The three experiments used in this study. (a) In experiment 1, we constructed artificial nests with two eggs and placed nests at low density, high density, or in a gull colony. (b) Photo of an artificial nest with one coot and one duck‐type egg. (c) In experiment 2, we placed painted eggs in active nests of three common species of hosts. (d) A red‐gartered coot nest with a real duck egg for comparison with the painted eggs used in artificial nests. Experiment 3 was nested within the other two experiments (a, c) and comprised adding either two brown coot‐like eggs or one brown and one white duck‐like egg to each artificial nest (a) or, adding either one white duck‐like egg or one host‐like egg (brown for coot, green for gull) into an active nest attended by the host (c). Host drawings by Hilary Burn (coots) and Ian Lewington (gull) (copyright Lynx Edicions).

Here, we test several key assumptions of this “bodyguard” hypothesis for the evolution of brood parasitism in the black‐headed duck using three sets of field experiments (Figure 2). First, we created artificial nests with experimental eggs to assess the background levels of predation pressure in the absence of parental defense on the same Argentine wetlands where we conducted breeding studies of black‐headed ducks (Lyon & Eadie, 2004, 2013). Second, we added similar types of experimental eggs to active host nests to compare the mortality rates of eggs in tended versus untended nests and hence determine the magnitude of the effect that three main host species have on the survival of eggs in their nests. Third, we compared predation rates on eggs that were similar in color to hosts and relatively cryptic to those of white eggs similar to ducks, to evaluate the possibility that white duck eggs might instead attract predators to host nests and thereby obviate some or all of the bodyguard benefits. Our experimental design nested the egg treatment (host or white duck‐colored eggs) within each of the above two nest type experiments (artificial nests and active host nests; Figure 2).

Not included in our study is an assessment of the survival rate that ducks would have were they to nest on their own; this counterfactual is impossible to test directly because black‐headed ducks never have their own nests. We can compare nest success of duck eggs in other species of ducks that nest in similar marshes, but such comparisons are limited by the paucity of data and potential differences in nest habitat or parental behavior. Moreover, most species of open water marsh‐nesting ducks do not aggressively defend their nests and so direct comparisons would be questionable. Accordingly, our study instead focuses on testing the three critical assumptions underlying the body guard hypothesis: specifically, (1) the risk of nest predation in these marshes is extremely high in the absence of aggressive parenting; (2) the main host species are sufficiently aggressive as nest defenders that duck eggs laid in their nests experience high survivorship despite the high potential risk for egg mortality; and (3) the presence of white duck eggs does not lead to an elevated predation risk of those eggs nor of the parasitized host nest.

## 2 METHODS

We conducted the experiments on two wetlands southwest of General Lavalle, Buenos Aires Provence, Argentina in late October and early November 1994 (map location shown in Lyon & Eadie, 2013). Black‐headed ducks parasitize a variety of hosts in these wetlands, but three species dominate and account for 92% of all parasitism: red‐gartered coots (*Fulica armillata*), red‐fronted coots (*Fulica rufifrons*), and brown‐hooded gulls (*Larus maculipennis*) (Lyon & Eadie, 2004, 2013). All three species construct large conspicuous nests over the water in the dominant vegetation found in all the wetlands, the bulrush *Schoenoplectus californicus*. Typically, nests are built in fairly sparse vegetation or at the edge of moderately dense vegetation, and they are usually conspicuous from a distance. Some red‐fronted coot nests are built in denser vegetation but are still visible from above. Therefore, nest crypsis is unlikely to be a major factor that determines whether nests are depredated, particularly by avian predators that search for nests by flying at low heights over the marshes. Instead, parental care—specifically aggressive nest defense—is likely to be important. Our experiments allowed us to test this assumption. Our natural history observations during nest surveys suggested that chimango caracaras (*Milvago chimango*) are probably the most important avian predator on nests, but other possible avian nest predators that breed in these wetlands include southern caracaras (*Caracara plancus*) and long‐winged harriers (*Circus buffoni*). Rarely, we observed the lutrine opossum (*Lutreolina crassicaudata*), a mammalian predator, preying on both adults and eggs of the hosts at our study area (B. Lyon, unpublished data).

### 2.1 Artificial nest experiments

We used artificial nests containing painted chicken eggs to evaluate nest survival rates in the absence of parental defense. This experiment had two treatment levels: nest setting and type of eggs present. There were three nest setting treatments: (1) high nest density, (2) low nest density, and (3) nests placed within an active brown‐hooded gull colony. Nest density has been found to affect nest predation rates in experimental nest studies (Major & Kendal, 1996) so we assessed the effect of nest density with two transects that bracketed the ranges of natural densities in the two species of host coots in the study wetlands. We conducted the gull colony treatment because the projective benefits of nesting near aggressive species are well‐known (Haemig, 2001; Quinn & Ueta, 2008) and have been previously demonstrated specifically for brood‐hooded gulls in Argentina (Burger, 1984).

The artificial nests were placed in habitat used by the hosts—areas of open water with patches of bulrush (Figure 2b). We chose a range of vegetation densities so that our experimental nests approximately matched the range of densities of stems in patches used as nest sites by actual hosts. To make each artificial nest, a 20 cm × 20 cm poultry wire mesh basket was attached with wire to the stems of a cluster of a bulrush plant, close the water (roughly 0.3 m above the water; Figure 2b). We then constructed a nest in the basket using the same dead bulrush stems that the coot hosts use to construct their nests.

Each of the three nest setting treatments comprised 20 nests, divided equally between the two egg treatments: 10 “unparasitized” nests containing two chicken eggs painted to resemble coot eggs and 10 “parasitized” nests with one chicken egg painted to resemble a coot egg and a second chicken egg painted white to resemble a black‐headed duck egg (Figure 2a,b). We used two eggs in each nest because this was the minimum number that enabled our two egg treatments and minimized the large number of eggs that had to be painted for our large‐scale experiment (120 eggs total in the artificial nest experiments). Spectrophotometer readings indicated that the latex paint matched the natural wavelengths of real coot and duck eggs with the exception that the painted eggs did not reflect in the UV wavelengths. To the human eye, the parasitism treatment resembled parasitized nests of the most important host, the red‐gartered coot (Figure 2b vs. Figure 2d).

In the “low‐density” treatment, artificial nests were placed 100 m apart along a transect on the wetlands at Estancia Palenque, and the experiment began on 30 October 1994. The two treatments (parasitized and unparasitized) were alternated along the transect. In the “high‐density” treatment, established on a different part of the same large wetland on 6 November 1994, nests were placed 30 m apart, also in a transect. Finally, the “gull colony” treatment was established in an active brown‐hooded gull colony at Estancia Cari Lauquen on 8 November 1994. To permit all nests to remain within the perimeter of the gull colony, nests were not set in a transect but placed haphazardly within the colony. We did not measure the density of the gull nests, but visual assessment indicated that the gull nests were spaced at lower densities (often 10–20 m apart) than is typical for high‐density colonial gulls nesting on dry land. All nests were checked every one to three days. We checked the high‐ and low‐density nests for a total span of seven and eight days, respectively, and the gull colony nests for a total span of 6 days. The experiments were done on the same large wetland complex but far enough apart so that they would not influence each other. These marshes are interconnected and cover extensive areas. We attempted to separate our experiments spatially (the two treatments at Estancia Palenque were ~1–2 km apart, the gull colony at Estancia Cari Lauquen was ~12 km away) so that one set of experiments did not influence another, but the predator and host communities were the same.

### 2.2 Experimental egg additions to active host nests

The second experiment, with active host nests, was conducted in the same year (1994) at the Estancia Cari Lauquen wetlands. This experiment was undertaken primarily to study natural selection on egg mimicry (Lyon & Eadie, 2004) but it also allows us to compare the survival rates of the painted eggs in our experimental nests to the survival of similar painted eggs in active real host nests, and hence determine the degree to which host parental care improves the survival of eggs. As with the artificial nest experiment, this experiment had two levels of treatment: nest type, namely, species of host (red‐gartered coot, red‐fronted coot, brown‐hooded gull), and egg type, namely, a white duck‐like egg or an egg with the same background color as the host eggs. The experimental eggs were painted with the same type of paints as the artificial nest experiment eggs but the pattern and color differed for the egg treatments; unlike the artificial nests, the coot‐like eggs lacked spots and for the gull nests we added chicken eggs painted green to resemble the gull eggs (see figure 2a in Lyon & Eadie, 2004 for photographs of these eggs for the two coot hosts). In our previous study, we examined egg rejection rates; here, we examined survival rates of the nests treated with these painted eggs. The birds at these nests rejected many of the experimental eggs (particularly the two coot species) and we ceased monitoring any nests for survival after the experimental eggs were rejected. We monitored the survival of some nests for up to three weeks, but we limited our survival analysis here to the 10‐day period after the experimental eggs were added to ensure a time span roughly the same length as for the artificial nest experiments.

### 2.3 Assessing the effect of conspicuous duck eggs on nest predation

The two egg‐type treatments nested within both the artificial nest experiment and the active host nest experiment comprise a third separate experiment that addresses the specific effect of egg type on nest predation. We contrasted two treatments within each of the experimental groups described above: (1) nests with chicken eggs painted to match the eggs of hosts simulating an unparasitized nest and (2) nests with one host‐like egg and one white duck‐like egg simulating a parasitized nest (Figure 2). For the artificial nests without parental care, we considered only coot‐like eggs (brown) and duck‐like eggs (white), whereas in the second experiment with active host nests, we added brown coot‐colored eggs to coot hosts, and greenish‐colored painted eggs (mimicking gull eggs) to brown‐hooded gull nests (Figure 2c). These egg‐type treatments served as a third experiment that allowed us to test whether parasitized nests with the presence of a conspicuous white egg would be more likely to be discovered and depredated by a predator, and thereby reduce the advantage of parasitism as a means to avoid nest predation.

We used Cox proportional hazard survival models in JMP (JMP 15: 2020) to compare nest survival rates across experiments and treatments. Nests that survived to the end of each experiment were classified as right censored. For the artificial nest experiments, we included experiment and nest treatment as model effects. Partial predation, where a predator only took one of the two eggs on a given day, was common in the artificial nest experiment so we examined nest survival in two ways: (1) considering partial predation as predation (i.e., nests with partial predation were considered depredated) and (2) only counting nests that had lost both eggs as depredated. For the comparison of artificial nests and active host nests, we did not compare the two experiments but instead compared all the treatments in the two experiments combined.

## 3 RESULTS

### 3.1 Experiment 1—Nest and egg mortality differed among artificial nest experiments

**FIGURE 3:**
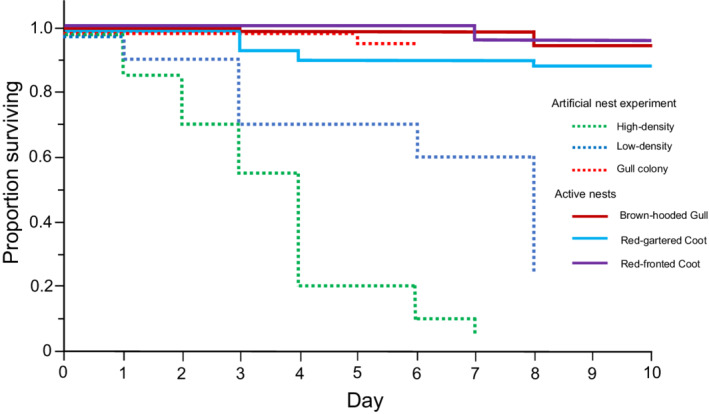
Survival of experimental eggs in three artificial nest experiments (dashed lines) and in active host nests of three species (solid lines). Survival times were based on when a nest lost all of its eggs; nests suffering partial predation (or egg rejection in real host nests) were considered active. All nests start at 1.0 on day 0 but are jittered to allow specific treatments to be more easily followed. Sample sizes: 20 nests for each of the artificial nest treatments: for active host nests, 69 red gartered coot nests, 72 brown‐hooded gull nests, 30 red‐fronted coot nests.

**FIGURE 4:**
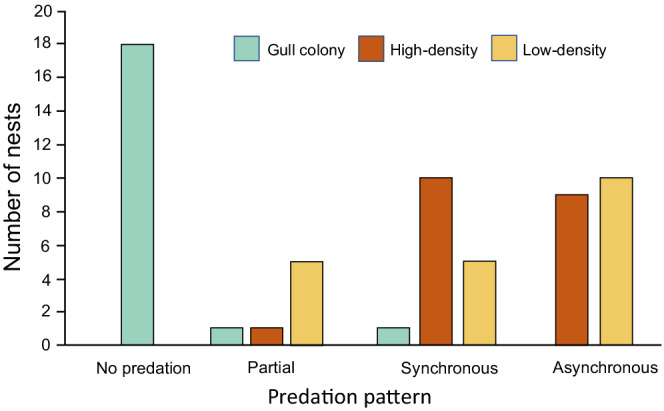
Egg predation patterns in the three artificial nest experiments. Partial predation indicates nests that lost only one of the two experimental eggs during the experiment. Synchronous nests lost both eggs in the same interval while asynchronous nests lost each of the two eggs in different intervals.

The survival rates differed across the artificial nest setting treatments (Table 1, Figure 3). The experimental nests suffered almost no predation in the gull colony treatment, but eggs were depredated very rapidly in both the high‐ and low‐density coot nest experiments (Figure 3). All nests in the high‐ and low‐density experiments suffered either partial or complete predation by the time we ended the experiment, with the majority experiencing complete predation (Figure 4). All three treatments differed in survival rates based on time to complete predation (risk ratio analysis: high vs. low *p* = .0022, high vs. gull *p* < .0005, low vs. gull *p* = .036). For survival based on the timing of the loss of any eggs (i.e., the first egg in nests with either asynchronous or partial predation or all nests with complete synchronous loss), the high‐ and low‐density coot nest experiments no longer differed but both differed from the gull colony treatments.

**TABLE 1:** Results of a Cox proportional hazard survival analysis comparing the survival rate of painted eggs in artificial nests with two levels of treatments.

*Whole model*				
Model	−LogLikelihood	df	Chi^2^	*p*
Difference	16.27	3	32.53	<.0001
Full	108.65			
Reduced	124.91			

*Effect likelihood ratio tests*			
Source	df	Chi^2^	*p*
Nest treatment	2	31.91	<.001
Egg treatment	1	1.07	.30

*Note*: The nest treatment (level 1) contained three nest situations: Low density, high density, and in a gull colony. The egg treatment (level 2, nested within level 1) contained two egg treatments: nests with two coot‐like eggs and nests with one coot‐like egg and one white duck‐like egg (Figure 1). Survival time was time to loss of both eggs.

For the nests that lost one or both eggs to predators, the most common pattern was asynchronous loss (loss of the two eggs occurred on different days) but synchronous loss was also quite common (both eggs disappear between the same sequential visits) (Figure 4). In contrast, partial predation was rare, and most nests had lost both eggs by the end of the experiment.

### 3.2 Experiment 2—Survival of active host nests with experimental eggs is very high

The survival rates of experimental eggs in active real host nests were uniformly high (Figure 3), and there were differences in survival rates compared to the artificial nest experiment (Table 2). Specifically, pairwise risk ratio comparisons of all the individual nest treatments for both the artificial and active host experiments revealed that survival in real host nests differed significantly from the low‐ and high‐density artificial nest experiments, but not from the artificial nest experiments in the gull colony (Appendix S1).

**TABLE 2:** Results of a Cox proportional hazards survival analysis comparing survival rates of painted eggs in the three nest situation treatments in the artificial nest experiment and the three host species treatments in the active host nest experiment.

*Whole model*				
Model	−LogLikelihood	df	Chi^2^	*p*
Difference	51.67	5	103.3	<.0001
Full	202.31			
Reduced	253.98			

### 3.3 Experiment 3—Conspicuous duck eggs do not increase the risk or timing of predation

The presence or absence of a duck‐like egg had no effect on the survival rate of nests in the artificial nest experiment (Table 1, effect of egg treatment). Also, there was no pattern of the duck eggs being lost first when the loss of eggs occurred asynchronously: in the 14 nests that lost eggs asynchronously, 6 nests (43%) lost the duck‐like egg first (binomial test, *p* = .79). Similarly, there was no effect of the presence of a duck‐like egg on nest survival in the active tended host nest experiment (Table 3).

**TABLE 3:** Results of a Cox proportional hazard survival analysis comparing the survival rate of painted eggs in active host nests with two levels of treatments.

*Whole model*				
Model	−LogLikelihood	df	Chi^2^	*p*
Difference	2.93	3	5.87	.12
Full	76.63			
Reduced	79.57			

*Effect likelihood ratio tests*			
Source	df	Chi^2^	*p*
Host treatment	2	3.64	.23
Egg treatment	1	1.97	.16

*Note*: The host treatment (level 1) contained three host species. The egg treatment (level 2, nested within level 1) contained two egg treatments: nests with a single host‐like egg and nests with a single white duck‐like egg, as shown in Figure 1c.

## 4 DISCUSSION

We used experimental nest and egg studies to assess the novel hypothesis that high rates of nest predation may have played a role in the evolution of obligate brood parasitism in the black‐headed duck. In an environment with extreme risk of nest predation, a parasite that lays her eggs in the nest of aggressive host species could increase her hatching success above what she would obtain were she to attempt to care for the eggs herself. Our experiments were designed to test three key assumptions of the bodyguard hypothesis for the wetlands in Argentina that are home to the parasitic black‐headed duck: (1) background nest predation rates are very high in the absence of any parental care; (2) the three main hosts used by the ducks are aggressive nest defenders that can maintain high nest survival rates despite the high potential predation risk; and (3) conspicuous white parasite duck eggs do not attract more predators or reduce the benefits of using a bodyguard host.

Our experiments revealed that the background rate of nest predation in the absence of attending parents is indeed extremely high—all nests in the low‐ and high‐density artificial nest experiments lost at least one egg within 5 or 6 days, and most lost both eggs. This time frame is a fraction of the incubation period for the duck eggs, roughly 23 days (B. Lyon & J. M. Eadie, unpublished data). Interestingly, the eggs disappeared more rapidly in the high‐density plot compared to the low‐density plot as has been shown in several previous studied (Major & Kendal, 1996), perhaps a reflection of the searching behavior of nest predators after they first encounter a nest with eggs. In contrast to the artificial nests, survival of active nests of the three main hosts was very high. Moreover, eggs in untended artificial nests in a gull colony suffered almost no predation, confirming the protective shield effect shown previously for this species of gull (Burger, 1984). The fact that all these experimental nests—artificial and active host nests alike—received painted experimental eggs rules out any confounding effect of natural versus painted eggs in driving the observed differences. Taken together, our experiments show that the potential for nest predation is extremely high in these Argentine marshes, but that the three main host species are very good at preventing predation, as Weller (1968) proposed. Additionally, the differences among the three treatments in the artificial nest experiment—more rapid predation of high‐density nests compared to low‐density nests, and the virtual absence of predation of nests in the gull colony—make good biological sense, which suggests that our experiments were biologically relevant and meaningful.

Because black‐headed ducks are obligate parasites, it is impossible to assess the counterfactual situation of how they would fare were to have their own nests. However, examining the nesting success of waterfowl species that nest in the same habitat can be informative. Our experiments were based on the assumption that overwater nesting is the relevant nest habitat for assessing the evolution of parasitism in the black‐headed duck given the phylogenetic placement of the black‐headed duck next to the stiff‐tailed ducks (tribe Oxyurini) (Livezey, 1995, 1997; Wuitchik et al., 2022), all of which are overwater nesters (Livezey, 1995). The rosy‐billed pochard (*Netta peposaca*), an overwater nesting species found in our study area in Argentina, provides the most comparable example of the nesting success expected for a nonparasitic duck in these wetlands. We observed only three pochard nests and did not follow them closely enough to determine predation rates. Weller (1968) followed the fates of six pochard nests, of which only one (16%) successfully hatched. This sample is admittedly too small to be anything other than suggestive, but if it is representative, it would indicate that a nesting duck might have a much higher success by laying her eggs in the nest of aggressive hosts like coots and gulls rather than tending the eggs herself. Further demographic work on nest success in rosy‐billed pochards would be worthwhile.

Studies of the nest mortality of facultative brood parasites could also be informative. Facultative interspecific brood parasites, like the redhead (*Aythya americana*), combine nesting with parasitism of other species. Facultative parasitism is thought to have been an intermediate stage in the evolution of obligate brood parasitism (Cichon, 1996; Lyon & Eadie, 1991) but is also clearly a stable evolutionary endpoint itself. Thus, comparing demographic attributes of the black‐headed duck to those of facultative parasites could provide insight into the factors that favor the transition from facultative to obligate parasitism. Nest survival rates for redheads are moderate and variable (overall 50%, considerably higher in populations with low levels of within‐species brood parasitism, which is a major cause of desertion; Woodin & Michot, 2020). Nesting success of their main host, the canvasback (*Aythya valisneria*) (Mowbray, 2020), is similar so there would be no selection for obligate brood parasitism from the enhanced hatching success with a ‘bodyguard’ host. Nonetheless, an interesting follow‐up to our study would be to conduct experimental nest predation studies in these northern hemisphere marshes and compare baseline predation rates with the survival rates of redhead and canvasback nests.

Our analysis of the success of active nests of the three host species—the two coots and the gull—is only meaningful if these species are important to the fitness of the parasite. At our site, 92% of ducks' eggs are laid in nests of these three hosts (Lyon & Eadie, 2013) and 83% of all ducklings hatch in nests of the two coot species (Lyon & Eadie, 2004). Detailed studies of host use have not been conducted elsewhere but it is worth noting that indirect evidence suggests that the two coot species also appear to be key hosts in other parts of the geographic range of black‐headed ducks (Cofré et al., 2007; Lyon & Eadie, 2013).

One countervailing selection pressure—rejection of duck eggs by some hosts—counteracts the benefits to parasites of high nest success in the hosts studied here. The two coot species reject a substantial fraction of duck eggs (Eadie & Lyon, 2020; Lyon & Eadie, 2004; Weller, 1968). Egg rejection is typically considered an evolutionary response to the costs of brood parasitism (Davies, 2000; Rothstein, 1990), in which case parasitism would have evolved before any egg rejection occurred. If this were the case, the high nest success of the hosts could have initially favored the evolution of parasitism, followed by a secondary reduction in parasite success once egg rejection evolved. However, egg rejection could have already been in place when the brood parasitism evolved—various lines of evidence suggest that egg rejection is a response to within‐species brood parasitism by the coots themselves, and not in response to parasitism by the ducks (Lyon & Eadie, 2004). Thus, considering only the survival benefits to ducks from the use of these aggressive hosts may present an overly optimistic view of the benefits of nest predation and nest survival as drivers of the evolution of brood parasitism. However, even with incidental egg rejection by hosts, the enhanced survival of nonrejected parasite eggs in the nests of aggressive hosts could mitigate the costs of rejection.

A second important finding from our experiments was that the presence of white duck eggs did not result in higher predation rates of host nests. Parasitic eggs and chicks that differ from those of the host can potentially bring costs or benefits to the hosts in terms of risk of nest predation (Canestrari et al., 2014; Mason & Rothstein, 1987; Wallace, 1889). The observation that coots reject some duck eggs but not all, despite the fact that most seem capable of recognizing the parasitic eggs (Eadie & Lyon, 2020), raises the possibility that the presence of a conspicuous duck egg might sometimes benefit hosts. One possibility is that the conspicuous white eggs serve as decoys that are more likely to be taken by a predator in cases of partial nest predation. Our treatments allowed us to assess this possibility, but we found no evidence of either predation benefits or costs to having a conspicuous white duck egg in a nest with otherwise cryptic eggs. Nests with duck eggs did not differ in survival compared to nests without duck eggs, in both the artificial and active host nest experiments. Moreover, in the artificial nest experiment, where we assessed patterns of partial predation in nests that had both a coot‐ and duck‐type egg, there was no difference in which egg type was taken first when eggs were lost eggs asynchronously (i.e., partial predation on the first day of predation).

The limitations of using artificial nests to assess naturally occurring levels of nest predation at active nests are well known (Butler & Rotella, 1998; Major & Kendal, 1996; Pärt & Wretenberg, 2002; Zanette, 2002). Our results did reveal a striking difference between artificial and active real nests but instead of being a liability of our experimental approach, investigating the difference in nest success of attended and unattended nests was a key point of our study. By design, we directly compared the same habitats and nest structures and used painted eggs for both our artificial and real host nests experiments; accordingly, the only difference was the presence or absence of an attending host. Nonetheless, there are still limits to what the experiments tell us because the specific type of wetland habitat used by the hosts, and explored by our experiments, may differ from those that a nesting duck would use (the counterfactual we can never know). The three host species all nest in areas with moderate to sparse vegetation, which makes their nests conspicuous—because these species aggressively defend their nests, they may not need to hide their nests. In contrast, ducks may hide their nests in denser patches of vegetation where they are more cryptic; the three pochard nests we found were better hidden than most coot and gull nests.

Our experiments indicate that nest predation could have played a role in the evolution of obligate brood parasitism from parental care in black‐headed ducks. Nest predation is thought to be an important driver of many aspects of avian life history (Martin, 1987, 2004) but has not previously been implicated in the evolution of brood parasitism other than early ideas that brood parasitism could be favored by spreading the risk of predation on eggs (Payne, 1977) or that it served as a proximate factor that triggers laying eggs in the nests of other birds (Hamilton & Orians, 1965). The fitness benefits of risk‐spreading and spatial bet‐hedging have been shown to be minimal (Bulmer, 1984; Hopper et al., 2003). In contrast to birds, links between predation and brood parasitism have long been thought to be important in fish. Sato (1986) reported obligate parasitism in a catfish that parasitizes mouth‐brooding parental care of its host cichlid, a form of parental care that reduces predation‐driven mortality of the fry. Facultative interspecific nest parasitism has been reported in several other fish and, to date, studies suggest that the main benefit to the parasitic offspring in these species is protection from predation (Baba et al., 1990; Johnston, 1994; McKaye, 1985; Wisenden, 1999).

Our study focused on the possible role of nest predation and hatching success in the evolution of obligate brood parasitism. We are not arguing that improved hatching success alone drove the evolution of obligate parasitism in the black‐headed duck but instead that it could have been an important contributing factor. We note that increases to other key demographic variables that result from abandoning parental care—fecundity, duckling survival, and adult survival—could all contribute to favor the evolution of obligate parasitism in black‐headed ducks (Eadie & Lyon, 2020; Lyon & Eadie, 1991, 2008). Based on our current understanding of waterfowl biology, some of these are more likely than others. Increased fecundity is unlikely to have played a dominant role in precocial birds, unlike that suspected for altricial birds (Lyon & Eadie, 1991); nonetheless, emancipation from parental care should in principle result in some increase in total fecundity given that the large amount of time required to incubate eggs and tend ducklings would be freed up to produce additional eggs. Conversely, if hosts at a suitable stage for successful parasitism are scarce, host availability could limit realized fecundity below the maximum potential for a brood parasite. For example, in a detailed study of marked female common cuckoos (*Cuculus canorus*), Wyllie (1981) found that cuckoos parasitizing reed warblers (*Acrocephalus scirpaceus*) laid on average just eight eggs per season, less than the clutch size of many parental species that raise two broods per season. The fecundity of black‐headed ducks remains to be studied, although genetic analysis of maternity patterns from one year of our study suggests that female black‐headed ducks do in fact have a very low fecundity, at least as assessed on a local scale (within our study site) (J. M. Eadie, B. E. Lyon, M. L. Jones, & M. R. Miller, unpublished data).

In some northern hemisphere waterfowl, adult female mortality on the nest is the major source of annual mortality (Arnold et al., 2012). For such species, emancipation from the dangers from nesting would bring a large fitness benefit via increased adult survival. However, most estimates of female mortality in northern hemisphere waterfowl come from upland nesting species where the high mortality of nesting hens is due to mammalian predators like foxes (*Vulpes vulpes*). Foxes do not hunt over water, and accordingly mortality of overwater nesting ducks by foxes is very low (Sargeant et al., 1984). For the South American wetlands, the impression we obtained from 18 person‐months searching the wetlands is that there are very few mammalian predators that pose a risk to an adult duck nesting overwater and that the abundant avian predators are a threat to nests but not adults.

The super precocial black‐headed ducklings are virtually unique among birds in receiving no parental care whatsoever after hatching (Lyon & Eadie, 2013; Weller, 1968), raising the possibility that juvenile survival could be a critically important life stage. The parameters we investigated here—nest predation and benefits from parasitizing successful hosts—should not affect survival after the ducklings leave the host nest. However, obligate brood parasitism would not be viable as a reproductive strategy if duckling survival were too low. As such, aspects of post hatching behavior could be an important prerequisite for the evolution of obligate brood parasitism. Although the offspring of most duck species feed themselves, the parent(s) play an important role in reducing mortality of the offspring (Eadie & Lyon, 1998). Intriguingly, the offspring of some stiff‐tailed ducks, close relatives of black‐headed duck, are tended by their parents for considerably shorter periods than those other groups of ducks, suggesting a tendency for more independent chicks that could successful rear themselves (Brua, 2020). Radio tagging studies have been used to assess survivorship of ducklings in various ducks (Korschgen et al., 1996) and could be invaluable for assessing survival of black‐headed ducklings, particularly with modern advances in tagging technology (Kays et al., 2015). In addition, a tagging study could confirm the suspicion that the ducklings are active at night (Lyon & Eadie, 2013), a behavioral trait that could be important for reducing the risk of predation, particularly by the abundant diurnal raptors like chimango caracaras.

Much previous work on the evolution of brood parasitism has focused on enhanced fecundity and/or survival of the brood parasitic adult, while little work has considered the role of nest and egg predation as an alternative driver. Our results suggest that the benefits of enhanced survival of parasite eggs through aggressive defense by “bodyguard” hosts clearly warrants further attention. It is possible, of course, that such benefits may only apply in some areas, such as the marshes in Argentina, where nest predation is extremely high. Conceivably, this could be the reason that there is only a single case of evolution of obligate brood parasitism in a precocial bird, the black‐headed duck, an idea that Milton Weller (1968) first suggested many years ago.

## AUTHOR CONTRIBUTIONS

**Bruce E. Lyon:** Conceptualization (lead); data curation (lead); formal analysis (lead); funding acquisition (lead); investigation (lead); methodology (lead); project administration (lead); resources (equal); supervision (lead); writing – original draft (lead); writing – review and editing (lead). **Alejandra Carminati:** Investigation (lead); methodology (equal). **Geneviève Goggin:** Investigation (lead); methodology (lead). **John M. Eadie:** Conceptualization (lead); data curation (equal); formal analysis (equal); funding acquisition (lead); investigation (lead); methodology (lead); project administration (equal); writing – original draft (lead); writing – review and editing (lead).

## FUNDING INFORMATION

This work was supported by the National Geographic Society (grant #5099‐93), the British Broadcasting Corporation (D. Attenborough's Life of Birds project), the Dennis G. Raveling Endowment of the University of California, Davis (JME), and the Univerity of California, Santa Cruz (BEL).

## CONFLICT OF INTEREST

The authors declare that they have no conflict of interest.

## Supporting information


Appendix S1

## Data Availability

The data that support the findings of this study are openly available in Dryad at https://doi.org/10.7291/D1PD7M.
