# Supplementary material for: Did extreme nest predation favor the evolution of obligate brood parasitism in a duck?
Source: Ecol Evol. 2022 Sep 19;12(9):e9251. doi: 10.1002/ece3.9251 (PMC9484301; doi:10.1002/ece3.9251)
Supplement: Supplementary file 1 — Appendix S1 [file ECE3-12-e9251-s001.pdf]

Appendix 1. Risk ratios for all 15 possible pairwise comparison of the main 6 experimental treatments.

Treatment 1	Treatment 2	Risk Ratio	$P > \chi^2$	Lower 95%	Upper 95%
Gull colony	High density	0.03	0.0008	0.0042	0.24
Gull colony	Low density	0.10	0.0248	0.012	0.74
Gull colony	BHG	1.79	0.61	0.19	16.54
Gull colony	RFC	2.69	0.49	0.17	43.90
Gull colony	RGC	0.75	0.79	0.09	6.19
High density	Low density	3.04	0.0038	1.43	6.45
High density	BHG	56.92	< 0.0001	18.15	178.43
High density	RFC	85.48	< 0.0001	11.12	657.20
High density	RGC	23.92	< 0.0001	9.77	58.61
Low density	BHG	18.73	< 0.0001	6.21	56.50
Low density	RFC	28.13	0.0012	3.72	212.98
Low density	RGC	7.87	< 0.0001	3.34	18.57
RFC	BHG	0.67	0.72	0.074	5.96
RFC	RGC	0.28	0.23	0.035	2.24
RGC	BHG	2.38	0.16	0.72	7.90

Summary of general patterns of differences or lack of differences in the above pairwise contrasts

	Other real hosts	Gull colony	Low-density	High-density
Real hosts	all $P > 0.15$	all $P > 0.49$	all $P \leq 0.002$	all $P < 0.0001$
Gull colony			$P < 0.025$	$P = 0.0008$
Low density				$P = 0.0038$
